# A Study on Bone Mass in Elderly Chinese Foot-Binding Women

**DOI:** 10.1155/2013/351670

**Published:** 2013-06-24

**Authors:** Yi Pan, Ling Qin, Mian Xu, Yin He, Juan Bao, Xian Guo, Jun Shu

**Affiliations:** ^1^Department of Endocrinology, The Second Affiliated Hospital of Kunming Medical University, No. 374 The Dianmian Avenue, Wuhua, Kunming, Yunnan Province 650101, China; ^2^Department of Orthopedics, Medical College of the Chinese University of Hong Kong, Shatin, New Territories, Hong Kong

## Abstract

The aim of this study is to understand the influences of the social custom of foot binding on female osteoporosis by means of comparing and analyzing the lumbar vertebrae and hip bone mass differences between the foot-binding aged women and unbound women of the same age at Qujing District of Yunnan Province. Of the examined people, 81.37% suffer from osteoporosis on the basis of lumbar vertebra (L1–L4) and femoral neck BMD, of which 82.14% for the foot-binding group and 80.44% for the unbound group. There is no statistical difference for the osteoporosis morbidity of the two groups. Compare the BMD value for various vertebrae, femoral neck, and rehabilitation of the two groups and find the BMD value for the other parts have no statistical difference except the BMD value of L1 centrum, which shows that foot binding does not significantly influence the overall bone mineral density of foot-binding women.

## 1. Introduction

Foot binding is a special custom from the ancient times to modern times; mainly for women of Han nationality, they use cotton and silk to swathe the feet of girls to make the front end sharp and restrict the free growth of both feet. With the gradual growth and development of feet, the arches of foot are extruded high and eventually form the special foot shape of “three-inch bound feet.” 

Foot binding violates the normal development of limbs but is also an important education, morality, behavior, beautifying, and life standard for Han women for thousands of years. With the changes and progress of times, modern civilization has thoroughly abandoned the deformity custom which binds women, the remaining foot-binding women in our society are gradually disappear with time going by, and the foot binding is going to meet its death, which has spread throughout China for thousands of years. To avoid the unique Chinese traditional custom disappearing with time passing by and to reserve more relevant social data of foot-binding behavior, we made a survey on the foot-binding women of Yunnan Region from the medical view. 

## 2. Data and Method

### 2.1. Exclusion Standard

Take inquiry for the selected foot-binding aged women and unbound women at Sanchahe Town, Luliang County, Qujing City, and Yunnan Province, to exclude diseases that may influence bone metabolism, such as liver and kidney disease, diabetes mellitus, thyroid and parathyroid disease, metabolic bone disease, ovariectomization, and subtotal gastrectomy; to exclude these with the medical history that they have been proved to have vertebral fracture and femoral fracture by X-ray examination in recent 3 months; to exclude these taking the drugs that may affect bone metabolism in recent 3 months, such as selective estrogen regulator, calcitonin, compound steroid hormones, bisphosphonate, thyroxine, parathyrin, or other antiosteoporosis drugs.

### 2.2. General Data

Totally 308 qualified testers are selected, who are all local countryside people of Han nationality. Of them, there are 204 foot-binding women, aged between 65 and 88, and averagely 76 and 104 unbound women, aged between 64 and 87, averagely 73. Select 102 healthy testers from the above 308 testers for bone mass and bone density measurement, of which 56 are from the foot-binding group, aged between 67–85, and averagely 74.8; 46 are from the unbound group, aged between 66 and 85, and averagely 72.5. 

### 2.3. Test Method

Measure the height, weight, waistline, and hipline of all testers and then, use body mass index and waist-hip ratio (WHR) for evaluation and comparative analysis. Bone mass and bone density measurement adopts GE-Lunar-Prodigy DXA for the lumber vertebra (L1–L4) and hip examination.

## 3. Result

### 3.1. Foot-Binding Shape Comparison

**Figure 1:**
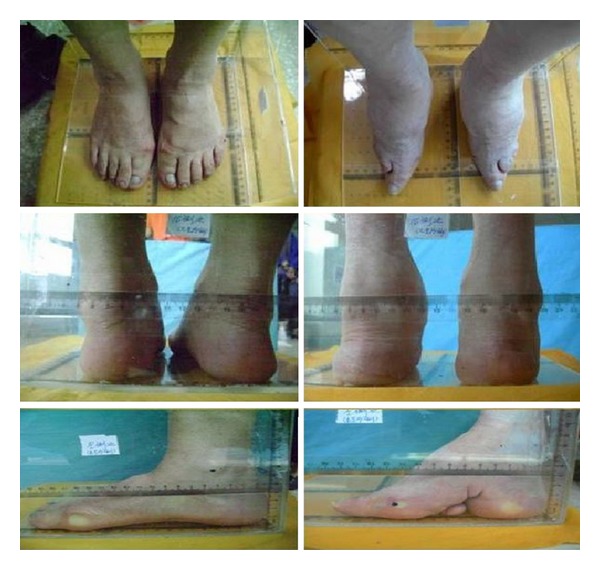
Comparison between the foot-binding deformity and normal foot.

**Figure 2:**
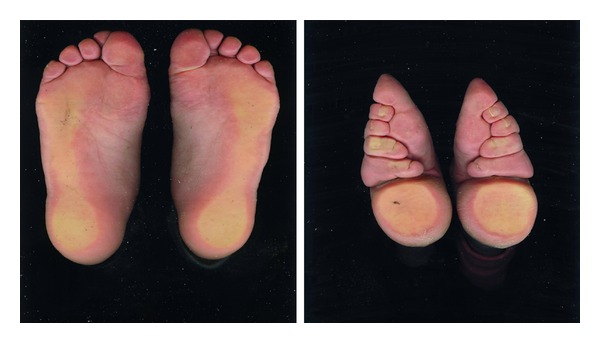
Comparison of pelma print scanning picture between the foot-binding deformity and normal foot.

By appearance-shape comparison between the foot-binding deformity and normal foot, the photo comparison of both feet of the testers is shown in Figure 1, and pelma print scanning picture comparison is shown in Figure 2.

From Figures 1 and 2, we can see that the appearance-shape characteristics of the foot-binding deformity are as follows: (1) foot is of triangle shape, with small front part which is similar to the top end of an awl, and large heel which is similar to a circular; (2) the 2nd–5th toes bend inwards at the pelma, the forefoot extrudes and draws close to the heel, there is a horizontal hollowing at the middle of the pelma, and the 5th toe is usually pressed in the hollowing; (3) arch of foot is extruded to rise, acrotarsium bulges upwards, and the forefoot has obvious plantar flexion; (4) after binding, the whole foot is obviously smaller than normal foot. 

### 3.2. General Health Condition Analysis

Divide the testers into the foot-binding group and unbound group and adopt the statistical method of group *T* examination to compare the differences in health index of the groups, such as height, weight, weight index, waistline, hipline, binocular vision, age of menopause, pregnancy and parity times, and breast-feed time. For results, please refer to Table 1. 

From Table 1, we can see that there is statistical difference in the weight, weight index, and pregnancy and parity times between the two groups (*P* < 0.05). The weight and weight index of the foot-binding group are lower than the unbound group, and the average pregnancy and parity times are higher than the unbound group.

Process the obtained height, weight, waistline, and hipline data from measurement and adopt weight index (BMI) and waist-hip ratio (WHR) for comparative analysis. BMI = weight (kg)/height (m)^2^; WHR = waistline (cm)/hipline (cm). Divide the testers into 4 groups according to BMI value as follows: thin: BMI < 18.5; normal: 18.5 ≤ BMI < 24; overweight: 24 ≤ BMI < 28; obesity: BMI ≥ 28; take WHR = 0.8 as the boundary to divide the testers into two groups, that is, noncentral obesity and central obesity, and adopt Chi-square test to compare the body shape differences between the foot-binding group and unbound group, and the result is shown in Table 2. Adopt the statistical method of group *T* examination to compare the BMI and WHR differences of the foot-binding group and unbound group, and the result is shown in Table 3. Adopt the grouping method according to ages to compare BMI and WHR differences of each age group. The age groups include the following: group below 70, group of 71–75, group of 76–80, and group above 81. After grouping according to ages, the comparison result of BMI and WHR of the foot-binding group and unbound group is shown in Table 4.

For the above data, we can see, in general, the foot-binding group is thinner than the unbound group, weight index is more ideal, but WHR has no statistical difference (Tables 2 and 3). After grouping according to ages, the comparison result of BMI and WHR of the foot-binding group and unbound group has no statistical difference, which shows that, foot binding does not influence the height, weight, waistline, or hipline of foot-binding women (Table 4).

### 3.3. Osteoporosis Morbidity Comparison

Take the lumber vertebra (L1–L4) or femoral neck BMD-*T* value as standard (take whichever is the lower of the both) for DXA examination testers, if *T* value is ≥ − 1.0, the bone mass is normal; if *T* value is between −1∼ − 2.5, it is osteopenia; if *T* value is ≤ − 2.5 or more, it is osteoporosis. The statistical result for the osteoporosis morbidity between the foot-binding group and the unbound group is shown in Table 7. The overall osteoporosis morbidity for all testers is 81.37%, of which 82.14% for the foot-binding group and 80.44% for the unbound group. Adopt Chi-square test to compare the osteoporosis morbidity between the two groups, and there is no statistical difference in the result (Table 5), which shows, the foot binding does not influence the osteoporosis morbidity for local aged women, but the aged women, especially rural women, have a high osteoporosis morbidity (Table 5).

### 3.4. DXA Result Analysis

Carry out DXA examination for L1, L2, L3, L4, L1–L4, femoral neck, and rehabilitation of 102 health testers and adopt the statistical method of group *T* examination to compare the difference between the two groups on BMC of each lumber interbody, BA, and BMD value of each lumber interbody, femoral neck, and rehabilitation (Table 6).

We can see that (1) for lumbar vertebra, the two groups have no statistical difference for L2 and L3 bone size but have statistical differences on BMD value of L1 vertebral body, and the other values have no statistical difference; (2) the whole lumbar vertebra (L1–L4) and femoral neck, rehabilitation BMD value, and *T* value have no statistical difference, which shows that the foot binding does not influence the overall BMD of the lumbar vertebra and hip of the foot-binding people, and the two groups have equivalent risks of lumbar vertebra and hip fracture.

Meanwhile, no matter for the foot-binding group or the unbound group, their bone mineral density is significantly lower than femoral neck, which shows that, for aged women, the risk of lumber vertebra fracture is higher than that of femoral neck (Table 7).

As the morbidity of osteoporosis has clear relevance with age [1, 2], so age grouping is adopted for further comparison. The age groups include the following: group below 70, group of 71–75, group of 76–80, and group above 81, with statistical method of independent sample *t* examination. The result is shown in Table 8.

From the results in Table 8, we can see, after grouping according to ages, the two groups have no statistical difference for lumbar vertebra *T* value and hip *T* value comparison, which shows that there is little difference in the BMD of the lumber vertebra and hip of testers of both groups, foot binding does not obviously influence the BMD of the lumber vertebra and hip of foot-binding people, and the two groups have equivalent risks of lumbar vertebra and hip fracture.

## 4. Discussion

### 4.1. Appearance-Shape Observation for Foot-Binding Deformity

By visual observation for photo of both feet and pelma print canning of the testers to compare and analyze the appearance shape of the foot-binding deformity and normal foot, we can find the appearance-shape characteristics of foot-binding deformity as follows: (1) foot is of triangle shape, with small front part which is similar to the top end of an awl, and large heel which is similar to a circular; (2) the 2nd–5th toes bend inwards at the pelma, the forefoot extrudes and draws close to the heel, there is a horizontal hollowing at the middle of the pelma, and the 5th toe is usually pressed in the hollowing; (3) arch of foot is extruded to rise, acrotarsium bulges upwards, and the forefoot has obvious plantar flexion; (4) after binding, the whole foot is obviously smaller than normal foot.

### 4.2. DXA Examination Result for Lumber Vertebra and Hip

It is theoretically predicted that, because of foot binding, with action difficulty and the limited scope of activities, they rarely take part in social activities and housework; thus, the bone mass of the lumbar vertebra and hip of foot-binding women should be obviously lower than that of unbound women.

According to the actual examination result, after grouping according to ages, there is no statistical difference for lumbar vertebra *T* value and hip *T* value of both groups, which shows that there is little difference in the BMD of the lumber vertebra and hip of testers of both groups, foot binding does not obviously influence the BMD of the lumber vertebra and hip of foot-binding people, and the two groups have equivalent risks of lumbar vertebra and hip fracture. It may be because the testers are all from rural areas; after foot binding, no matter what physiological differences they have, there is no obvious difference between labor opportunity and labor intensity of them. Especially for testers, at the youth age when the bone mass accumulates, they all need to take part in labor of the same intensity to make life, thus, making the influence of foot binding on body bone mass to the minimum. The result demonstrates again from another aspect that exercise and sports play an important role in accumulating peak bone mass and delaying bone mass declination [3–5].

Besides, L1 centrum BMD value of the foot-binding group is significantly lower than the normal people; maybe we can deduct that foot binding has little influence on the lower part of axial skeleton of human body because foot binding changes the way women walk. Foot-binding people walk with both heels down to the ground; the driving force is all on the muscle of the thigh and depends on the activity of knee joint. In this case, the muscle of hip and thigh is more developed, therefore, the lower part of lumbar vertebra always drives exercise, and BMD declination is not obvious. Whether foot binding influences BMD of the medium and upper part bone of the axial skeleton can be further confirmed by thoracic vertebra DXA examination.

### 4.3. Measuring Result of Weight Index and WHR

Current researches have proved that height and weight are important factors to influence BMD of human body [6]. WHR is the ratio between waistline and hipline, and also the index to predict obesity at early stage [7]. Theoretically, after foot binding, people shall always suffer from continuous pain, and their diet and sleep are both disturbed, causing great influence on their body growth and development. Therefore, the weight index of the foot-binding group is lower than the unbound group, and the testers of the foot-binding group are generally light weight group (BMI < 18.5).

Secondly, the 2nd–5th toes of the pelma of foot-binding people twine at the pelma, and the forefoot only has the halluces to carry the load, which changes the structure of 3 points of normal foot which carry the load. Besides, the foot arch on the pelma to buffer the force disappears, while walking, their heel falls down to the ground, and the strength of the thigh is used to take a step; eventually, thigh muscle is developed, calf muscle shrinks, and the stress is applied to the knee joint and hip joint until hip joint changes. As a result, after many years' foot-binding life, the hip line of the foot-binding people should be larger than that of the unbound people, and the WHR of the foot-binding people should be smaller than that of the unbound people. But the details for how the skeleton of hip joint is changed still need further comparison and analysis after double hip plain film examination.

From the measurement result, we can see that the testers from the foot-binding group are generally thinner than the testers from the unbound group, and their weight index is more ideal. But after grouping according to ages, there is no statistical difference in weight index and WHR of both groups, which shows that foot binding does not obviously influence the height, weight, waistline, and hipline of the foot-binding people. No matter they are from the foot-binding group or the unbound group, their weight index and WHR are basically within the ideal scope.

## 5. Conclusion

Although foot binding influences the walking posture and even life of the foot-binding people by means of changing foot shape, it has little influence on the bone mass of the whole body of the foot-binding people, except partial bones. The aged women of the rural area of Qujing District of Yunnan Province suffer from a high osteoporosis morbidity, and their risk for lumbar vertebra fracture is higher than the femoral neck.

## Figures and Tables

**Table 1:** General health indicators of normal foot group (*n* = 104) and foot-binding group (*n* = 204).

	Normal foot group ($\bar{x}\;\pm \;s$)	Foot-binding group ($\bar{x}\;\pm \;s$)	*t*	*P*
Age (Years)	72.95 ± 4.50	75.99 ± 4.58	−5.482	0.001
Height (cm)	149.32 ± 6.12	148.36 ± 6.03	1.302	0.194
Weight (kg)	49.03 ± 8.55	45.73 ± 7.33	3.485	0.001
BMI	21.95 ± 3.36	20.79 ± 3.19	2.928	0.004
Waistline (cm)	72.81 ± 7.92	72.37 ± 7.68	0.465	0.642
Hipline (cm)	85.55 ± 6.46	84.46 ± 5.60	1.512	0.131
Left vision	0.75 ± 1.14	0.55 ± 0.94	1.605	0.110
Right vision	0.74 ± 1.16	0.55 ± 0.96	1.465	0.144
Age of menopause (years)	47.49 ± 4.52	48.05 ± 3.72	1.160	0.247
Gravidity	6.77 ± 2.22	7.64 ± 2.86	2.689	0.008
Parity	6.21 ± 2.13	7.02 ± 2.63	2.724	0.007
Lactation month time	127.80 ± 58.35	125.57 ± 61.07	−0.304	0.762
Footbinding age (years)		8.39 ± 3.16		

**Table 2:** Bodily form contrast of normal foot group (*n* = 104) and foot-binding group (*n* = 204).

	BMI				WHR	
	Slants thin	Normal	Overweight	Fat	Noncentral obesity	Central obesity
Normal foot group (%)	15 (14.7)	61 (58.7)	25 (24.5)	3 (2.9)	54 (51.9)	50 (48.1)
Foot-binding group (%)	56 (27.5)	112 (54.9)	30 (14.7)	6 (2.9)	96 (47.1)	108 (52.9)
*χ* ^2^	8.605				0.472	
*P*	0.035				0.492	

**Table 3:** Comparison of BMI and WHR of normal foot group (*n* = 104) and foot-binding group (*n* = 204).

	Foot-binding group	Normal foot group	*T*	*P*
BMI	20.79 ± 3.19	21.95 ± 3.36	−2.928	0.004
WHR	0.856 ± 0.055	0.850 ± 0.052	0.859	0.391

**Table 4:** Body comparison according to age groups between normal foot group (*n* = 104) and foot-binding group (*n* = 204).

Grouping	BMI		*T*	*P*	WHR		*T*	*P*
	Foot-binding group	Normal foot group			Foot-binding group	Normal foot group		
Group below 70	21.64 ± 3.71	22.06 ± 2.92	−0.465	0.644	0.842 ± 0.065	0.847 ± 0.052	−0.325	0.747
Group of 71–75	21.75 ± 2.95	22.40 ± 3.62	−1.691	0.092	0.844 ± 0.052	0.857 ± 0.052	−1.335	0.184
Group of 76–80	20.47 ± 3.14	20.81 ± 2.97	−0.454	0.651	0.843 ± 0.050	0.835 ± 0.050	1.328	0.185
Group above 81	21.16 ± 3.48	22.66 ± 5.01	−0.858	0.396	0.878 ± 0.055	0.879 ± 0.060	−0.009	0.993

**Table 5:** Osteoporosis diagnosis rate between normal foot group (*n* = 46) and foot-binding group (*n* = 56).

	Normal	Osteopenia	Osteoporosis
Normal foot group	2.17%	17.39%	80.44%
Foot-binding group	3.57%	14.29%	82.14%
Whole	2.94%	15.69%	81.37%
*χ* ^2^			0.049
*P*			0.826

**Table 6:** Lumbar spine and hip DXA data analysis between normal foot group (*n* = 46) and foot-binding group (*n* = 56).

	Normal foot group	Foot-binding group	*T*	*P*
BMC g				
L1	6.870 ± 1.492	6.463 ± 1.576	−1.320	0.19
L2	7.717 ± 1.875	7.685 ± 1.763	−0.088	0.93
L3	9.247 ± 2.251	9.567 ± 2.255	0.708	0.48
L4	10.833 ± 2.469	10.951 ± 2.426	0.24	0.811
L1–L4	34.668 ± 7.307	34.401 ± 7.306	−0.182	0.856
BA cm^2^				
L1	9.768 ± 1.211	10.170 ± 1.217	1.653	0.101
L2	10.757 ± 1.446	11.329 ± 1.588	1.87	0.064
L3	12.049 ± 1.616	12.712 ± 1.532	2.109	0.037
L4	13.521 ± 1.999	13.809 ± 1.894	0.742	0.46
L1–L4	46.095 ± 4.940	47.735 ± 5.472	1.563	0.121
BMD g/cm^2^				
L1	0.702 ± 0.119	0.631 ± 0.113	−0.306	0.003
L2	0.713 ± 0.126	0.676 ± 0.114	−1.548	0.125
L3	0.764 ± 0.134	0.749 ± 0.135	−0.546	0.586
L4	0.805 ± 0.156	0.792 ± 0.137	−0.445	0.658
L1–L4	0.750 ± 0.126	0.718 ± 0.117	−1.317	0.191
Femoral neck	0.664 ± 0.092	0.647 ± 0.085	−0.939	0.35
Rehabilitation	0.737 ± 0.118	0.709 ± 0.095	−1.348	0.181
*T* value (L1–L4)	−3.02 ± 1.01	−3.257 ± 0.96	−1.206	0.231
*T* value (femoral neck)	−1.615 ± 0.96	−1.870 ± 0.79	−1.472	0.144

**Table 7:** The lumbar spine and femoral neck bone mineral density (normal foot group *n* = 46; foot-binding group *n* = 56).

	*T* value (L1–L4)	*T* value (femoral neck)	*T*	*P*
Normal foot group	−3.02 ± 1.01	−1.615 ± 0.96	6.839	0.000
Foot-binding group	−3.257 ± 0.96	−1.870 ± 0.79	8.349	0.000

**Table 8:** Comparison of *T* values of the lumbar spine and hip *T* values in the different age groups between normal foot group and foot-binding group.

Grouping	Lumbar *T* value		*T*	*P*	Hip *T* value		*T*	*P*
	Foot-binding group	Normal foot group			Foot-binding group	Normal foot group		
Group below 70	−2.94 ± 0.57	−2.96 ± 1.16	0.029	0.977	−1.73 ± 0.69	−1.40 ± 1.21	−0.669	0.511
Group of 71–75	−3.29 ± 1.07	−3.21 ± 1.00	−0.291	0.773	−1.85 ± 0.93	−1.84 ± 0.84	−0.020	0.984
Group of 76–80	−3.35 ± 1.01	−2.69 ± 0.84	−1.540	0.137	−1.93 ± 0.65	−1.44 ± 0.68	−1.672	0.108
Group above 81	−3.15 ± 0.61	−2.75 ± 0.21	−0.852	0.442	−1.98 ± 0.69	−1.55 ± 0.78	−0.692	0.527
